# First trimester sCD40L levels associated with adverse neonatal outcomes in euthyroid pregnant women with positive TPOAb

**DOI:** 10.3389/fendo.2023.1097991

**Published:** 2023-05-23

**Authors:** Xinxin Chen, Qingyao Wang, Huanhuan Zang, Xiangguo Cong, Qiong Shen, Lei Chen

**Affiliations:** Department of Endocrinology, The Affiliated Suzhou Hospital of Nanjing Medical University, Suzhou, China

**Keywords:** thyroid peroxidase antibody, pregnancy, adverse neonatal outcomes, sCD40L, platelet

## Abstract

**Backgrounds:**

It remained unclear whether isolated positive thyroid peroxidative antibodies (TPOAb) were associated with adverse maternal and neonatal outcomes. The purpose of this study was to observe adverse neonatal outcomes among euthyroid pregnant women with positive TPOAb and to investigate the underlying risk factors.

**Methods:**

Euthyroid pregnant women with TPOAb positivity were enrolled and followed up in our study. Adverse neonatal outcomes such as preterm birth, low birth weight, and fetal macrosomia were observed. Clinical data in the first trimester were collected and compared between groups with or without adverse neonatal outcomes. Maternal serum soluble CD40 ligand (sCD40L) was also measured at the same time.

**Results:**

A total of 176 euthyroid pregnant women with TPOAb positivity were finally enrolled and analyzed in our study. Thirty-nine (22.16%) euthyroid women with TPOAb positivity were found to have adverse neonatal outcomes. Thirteen participants received assisted reproductive technology (ART) in our study, and seven participants were in the adverse neonatal outcome group. Preterm birth, low birth weight, and fetal macrosomia were the most common comorbidities. The proportion of receiving ART and the levels of sCD40L and platelet were significantly higher in the adverse neonatal outcome group (all *P* < 0.05). Multivariate regression analysis showed that sCD40L and receiving ART were the independent risk factors for adverse neonatal outcomes. The odds ratio values of sCD40L higher than 5.625 ng/ml were 2.386 [95% confidence interval (CI) = 1.017 to 5.595; *P* = 0.046] for overall adverse neonatal outcome, 3.900 (95% CI = 1.194 to 12.738; *P* = 0.024) for preterm birth, and 3.149 (95% CI = 0.982 to 10.101; *P* = 0.054) for low birth weight.

**Conclusions:**

Approximately one of the four euthyroid women with TPOAb positivity might have adverse neonatal outcomes. Measurement of sCD40L in first trimester might have a predictive value for adverse neonatal outcomes in euthyroid pregnant women with positive TPOAb.

## Introduction

Thyroid hormones are important determinants for the development of fetuses, especially in early pregnancy (1). Thyroid autoimmunity (TAI), defined with the presence of thyroid peroxidative antibodies (TPOAb) or thyroglobulin antibodies (TgAb) in circulation, is the most common cause of thyroid dysfunction in women of childbearing age. In the worldwide, about 8%–14% women might have TAI disease (2). TAI in pregnancy could affect the stabilization of maternal thyroid hormones and is related to adverse outcomes for pregnant women and fetuses, such as gestational diabetes mellitus, pregnancy-induced hypertension, miscarriage, preterm birth, and mental development in offspring in previous epidemiological study (3, 4).

Isolated positive TPOAb women are particular phenotype that is defined as having normal thyroid function and positive TPOAb in circulation. Studies have shown that euthyroid women with TPOAb positivity were also associated with preterm birth, abnormal fetal growth, and so on (5–7). It was hypothesized that immune disorders played a central role in the mechanism of adverse pregnancy outcomes in these patients (8). The activation of autoreactive T cells and their related costimulatory molecules plays important roles in the immune response of TAI (9, 10). Thus, the changes in costimulatory molecules might be of special interest in euthyroid women with isolated positive TPOAb with adverse pregnancy outcomes.

As a pair of important costimulatory molecules, CD40 and its ligand, CD40L, were closely related to the production of antibodies (11). CD40L is expressed on the membrane of T cells and linked to CD40 on B cells, providing a signal for the differentiation, proliferation, and activation of B cells. CD40L exists in both membrane and soluble forms *in vivo*. Soluble CD40L (sCD40L) is produced by the shedding of CD40L and has similar functions to CD40L (12). sCD40L was found to be positively correlated with TPOAb (13). However, there is no study on the clinical significance of sCD40L levels in euthyroid women with positive TPOAb and its association with adverse neonatal outcomes. Given the early effects of TAI and low-grade systemic inflammation in pregnant women, we tried to determine whether there was a relationship between biomarkers of immune activation sCD40L levels in the first trimester and adverse neonatal outcomes.

The purpose of this study was to observe the incidence of adverse neonatal outcomes in euthyroid women positive for TPOAb and to investigate the association between maternal serum sCD40L levels or other underlying risk factors in early pregnancy and adverse neonatal outcomes.

## Methods

### Participants

The longitudinal observational study was conducted at the affiliated Suzhou Hospital of Nanjing Medical University during the period from March 2020 and July 2021. Pregnant women with positive TPOAb and normal thyroid function in the first trimester were enrolled in our study. Positive TPOAb was defined with serum TPOAb titers greater than the upper limit of the reference value. Normal thyroid function was defined with normal free thyroxine (FT4) and normal thyroid-stimulating hormones (TSH) in serum.

The 2017 American Thyroid Association (ATA) guideline advocates the use of population-based reference ranges of TSH during pregnancy, whereas if these ranges are not available, they recommended using 4.0 mIU/L as the upper reference limit of TSH for the first trimester. Thus, TSH values > 4 µIU/ml were excluded in our study to eliminate the possible effect of subtle thyroid dysfunction (14).

All participants enrolled in the first trimester and follow-up until childbirth. Inform consent was signed in each participant before the enrollment. Adverse neonatal outcomes (preterm birth, low birth weight, and fetal macrosomia) were recorded and analyzed.

**Figure 1:**
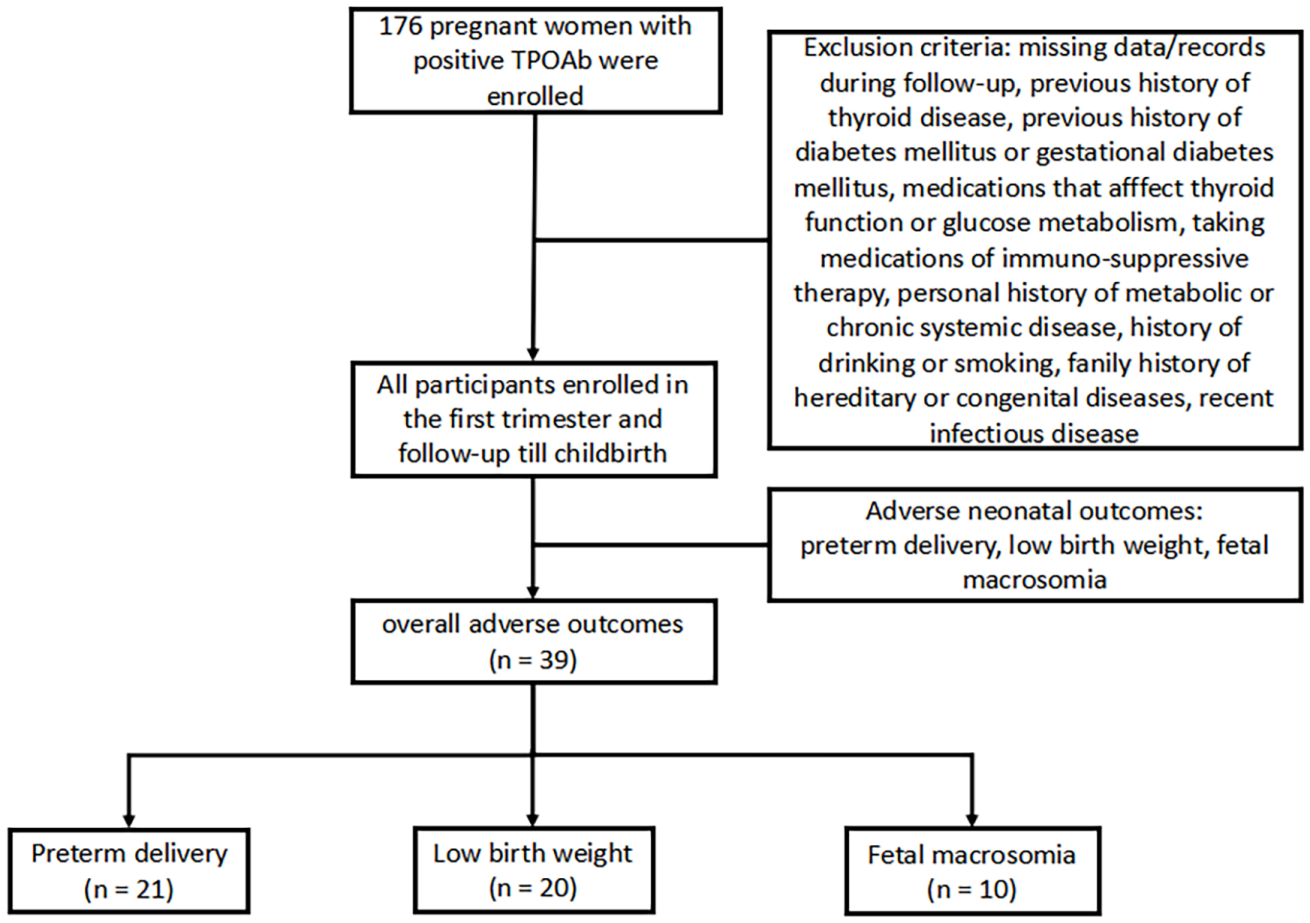
Flow diagram of study.

Premature birth was defined with newborns whose gestational age is less than 37 weeks but more than 28 weeks. Low birth weight was defined with newborns whose birth weight is less than 2,500 g. Fetal macrosomia was defined with a newborn with a birth weight of more than 4,000 g. The flow diagram of study was summarized in **Figure 1**.

The exclusion criteria for the participation in the study are as follows: age > 45 years old, missing data/records during follow-up, previous history of thyroid disease, previous history of diabetes mellitus or gestational diabetes mellitus (15), medications that affect thyroid function or glucose metabolism, taking medications of immuno-suppressive therapy, personal history of metabolic or chronic systemic disease, history of drinking or smoking, family history of hereditary or congenital diseases, and recent infectious disease.

### Data collection and laboratory measurements in early pregnancy

The demographical data of all participants such as age, gestational weeks, gravida, parity, pre-pregnancy body mass index (BMI), receiving assisted reproductive technology (ART) or not, type of ART such as in vitro fertilization (IVF), or intracytoplasmic sperm injection (ICSI) were recorded in the early pregnancy. All participants will undergo a routine obstetric examination in our hospital. Data of glycosylated hemoglobin A1c (HbA1c), fasting plasma glucose (FPG), ferritin, uric acid (UA), triglyceride (TG), total cholesterol (TC), high-density lipoprotein cholesterol (HDL-c) and low-density lipoprotein cholesterol (LDL-c), C-reactive protein (CRP), white blood cell (WBC), red blood cell (RBC), hemoglobin (Hb), and platelet were also collected.

Serum levels of TSH, FT4, and TPOAb of all participants in gestational weeks 10–13 were measured. Serum levels of TSH, FT4, and TPOAb were measured using automated chemiluminescent immunoassays (Architect i2000SR; Abbott Laboratories, Chicago, IL, USA). Reference ranges used in our study were as follows: TSH, 0.35~4.94 μIU/ml; FT4, 9.01~19.04 pmol/L; TPOAb, <12 IU/ml.

### Measurement of sCD40L

Fasting blood was collected in all participants in the early stage of pregnancy. Serum sample was store in −80°C refrigerator for centralized measurement. A commercial ELISA kit (Xvguang Kexing Antibody Biotechnology Co., Ltd.) was used to measure serum sCD40L. The minimum detectable dose was 0.015 ng/ml, with intra- and inter-coefficients of variation of <5%.

### Statistical analysis

Statistical analysis was performed using SPSS 26.0 and GraphPad Prism 8.0.2. The data were tested for normality using the Kolmogorov–Smirnov test. Data are presented as mean ± standard deviation for normally distributed variables or median (5% and 95% interquartiles) for nonnormally distributed variables. According to the adverse neonatal outcomes, the participants were divided into two subgroups. The differences between groups were compared by Student’s t-test or Mann–Whitney U-test. The Fisher’s exact test was used to test categorical variables. According to types of adverse outcomes, participants were divided into four subgroups. The differences among subgroups were performed with nonparametric test. Logistic regression analysis was carried out to evaluate the odds ratio (ORs) and 95% confidence interval (95% CI) of adverse neonatal outcome in euthyroid pregnant women with positive TPOAb. *P-*value of 0.05 or less was considered statistically significant.

## Results

### The comparison of baseline characteristics of pregnant women with or without adverse neonatal outcome

A total of 176 euthyroid pregnant women with positive TPOAb with complete data were finally enrolled and analyzed in our study. Thirty-nine of the 176 (22.16%) participants were having adverse neonatal outcomes. According to the occurrence of adverse neonatal outcomes, the participants were divided into groups: with adverse neonatal outcome (n = 39) and without adverse neonatal outcome (n = 137). The baseline and laboratory characteristics of the patients were provided in **Table 1**. There were no statistically significant differences between age, gestational weeks, pre-pregnancy BMI, gravida, parity, TSH, FT4, ferritin, HbA1c, FPG, UA, TG, TC, HDL-c, LDL-c, CRP, WBC, RBC, and Hb. Thirteen participants received ART were analyzed in our study. Six participants (five IVF and one ICSI) were in the group without adverse neonatal outcome, and seven participants (three IVF and four ICSI) were in the group with adverse neonatal outcomes. The proportion of receiving ART participants in adverse neonatal outcome group was higher than in the group without adverse neonatal outcome (*P* = 0.012, **Table 1**). However, there was no significant difference in the type of ART received (*P* = 0.265, **Table 1**).

**Table 1:** Baseline and demographical characteristics and laboratory parameters of women in the first trimester between normal and abnormal neonatal outcome groups.

	Abnormal neonatal outcome (n = 39)	Normal neonatal outcome (n = 137)	*P*-value*
Age (years)	29.74 ± 3.51	29.96 ± 4.04	0.766
Gestational weeks	12.83 ± 1.55	13.10 ± 1.93	0.429
Pre-pregnancy BMI (kg/m^2^)	22.73 ± 3.59	22.26 ± 2.96	0.410
Gravida	2 (1–3)	2 (1–2)	0.829
Parity	0 (0–1)	0 (0–1)	0.517
ART (n, %)	7 (17.95%)	6 (4.38%)	0.012*
IVF (n)	3	5	0.265
ICSI (n)	4	1	
TPOAb (IU/ml)	239.76 (88.7-771.36)	416.34 (161.07-642.84)	0.555
TSH (µIU/ml)	1.68 ± 0.69	1.60 ± 0.69	0.549
FT4 (pmol/L)	13.11 ± 1.40	12.74 ± 1.27	0.118
Ferritin (ng/ml)	48.96 (32.61–98.22)	54.49 (31.72–86.39)	0.763
HbA1c (%)	5.23 ± 0.22	5.19 ± 0.34	0.441
FPG (mmol/L)	4.56 ± 0.35	4.64 ± 0.46	0.362
UA (µmol/L)	218.39 ± 42.24	218.50 ± 52.48	0.990
TG (mmol/L)	1.57 ± 0.74	1.55 ± 0.68	0.837
TC (mmol/L)	4.86 ± 0.84	4.82 ± 0.79	0.795
HDL-c (mmol/L)	1.72 ± 0.35	1.80 ± 0.38	0.299
LDL-c (mmol/L)	2.56 ± 0.64	2.50 ± 0.67	0.585
CRP (mg/L)	2.84 (1.28–6.08)	2.29 (1.03–4.28)	0.412
WBC (× 10^9^/L)	8.47 ± 1.81	8.61 ± 1.77	0.655
RBC (× 10^12^/L)	4.26 ± 0.34	4.23 ± 0.37	0.609
Hb (g/L)	127.92 ± 8.19	128.31 ± 10.08	0.828
platelet (× 10^9^/L)	260.62 ± 62.51	225.91 ± 49.92	<0.001*
sCD40L (ng/ml)	8.28 ± 4.15	6.25 ± 2.43	0.005*

*P < 0.05 is statistically significant.

BMI, body mass index; ART, assisted reproductive technology; IVF, in vitro fertilization; ICSI, intracytoplasmic sperm injection; TPOAb, thyroid peroxidative antibodies; TSH, thyroid-stimulating hormones; FT4, free thyroxine; HbA1c, glycosylated hemoglobin A1c, FPG, fasting plasma glucose; UA, uric acid; TG, triglyceride; TC, total cholesterol; HDL-c, high-density lipoprotein cholesterol; LDL-c, lipoprotein cholesterol; CPR, C-reactive protein; WBC, white blood cell; RBC, red blood cell; Hb, hemoglobin.

Platelet and sCD40L were significantly higher in participants with the adverse neonatal outcome group (*P* < 0.001, *P* = 0.005). A positive correlation was found between sCD40L and platelet (*r* = 0.323, *P* < 0.001). There was no correlation between sCD40L and other parameters.

### Serum sCD40L and platelets were higher in early pregnancy in euthyroid pregnant women with adverse neonatal outcome

We then analyzed the types of adverse neonatal outcome that occurred. A total of 39 (22.16%) participants had adverse neonatal outcome including preterm birth (21, 11.93%), low birth weight (20, 11.36%), and fetal macrosomia (10, 5.68%).

**Figure 2:**
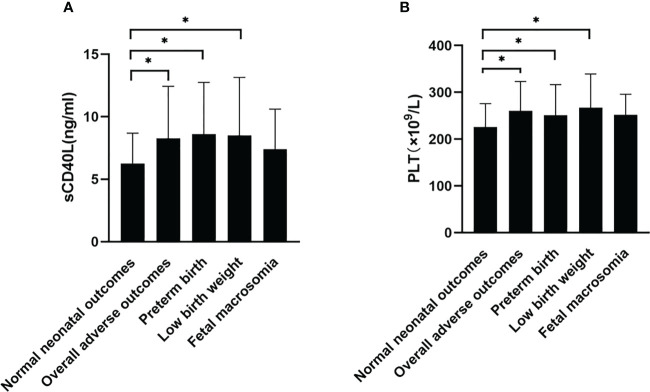
Comparison of sCD40L and platelet among groups with or without adverse neonatal outcomes. According to types of adverse neonatal outcomes, participants were divided into groups with normal neonatal outcome, overall adverse outcomes, preterm birth, low birth weight, and fetal macrosomia. sCD40L **(A)** and platelet **(B)** were compared among groups using one-way ANOVA test. *P < 0.05.

sCD40L levels of participants were 8.28 ± 4.15 ng/ml in the overall adverse neonatal outcome group, 8.60 ± 4.14 ng/ml in the preterm birth group, and 8.49 ± 4.65 ng/ml in the low birth weight group. sCD40L levels were found to be significantly higher in overall adverse outcomes, preterm birth, and low birth weight (*P* = 0.005, *P* = 0.019, and *P* = 0.047; **Figure 2A**). The sCD40L levels of participants with fetal macrosomia were 7.39 ± 3.22 ng/ml and were higher than those without in the adverse outcome group (6.25 ± 2.43ng/ml), but no statistically significances were found.

Platelet counts were also found to be significantly higher in overall adverse outcomes, preterm birth, and low birth weight (*P <* 0.001, *P* = 0.045, and *P* = 0.022; **Figure 2B**). There was no significant difference in the fetal macrosomia group. Twelve pregnant women were found to have preterm birth and low birth weight; sCD40L level was 8.43 ± 4.30ng/ml; platelet level was 246.42 ± 71.54 × 10^9^/L (data not shown).

### The cutoff value for sCD40L and platelet levels in early pregnancy in predicting adverse neonatal outcomes

**Figure 3:**
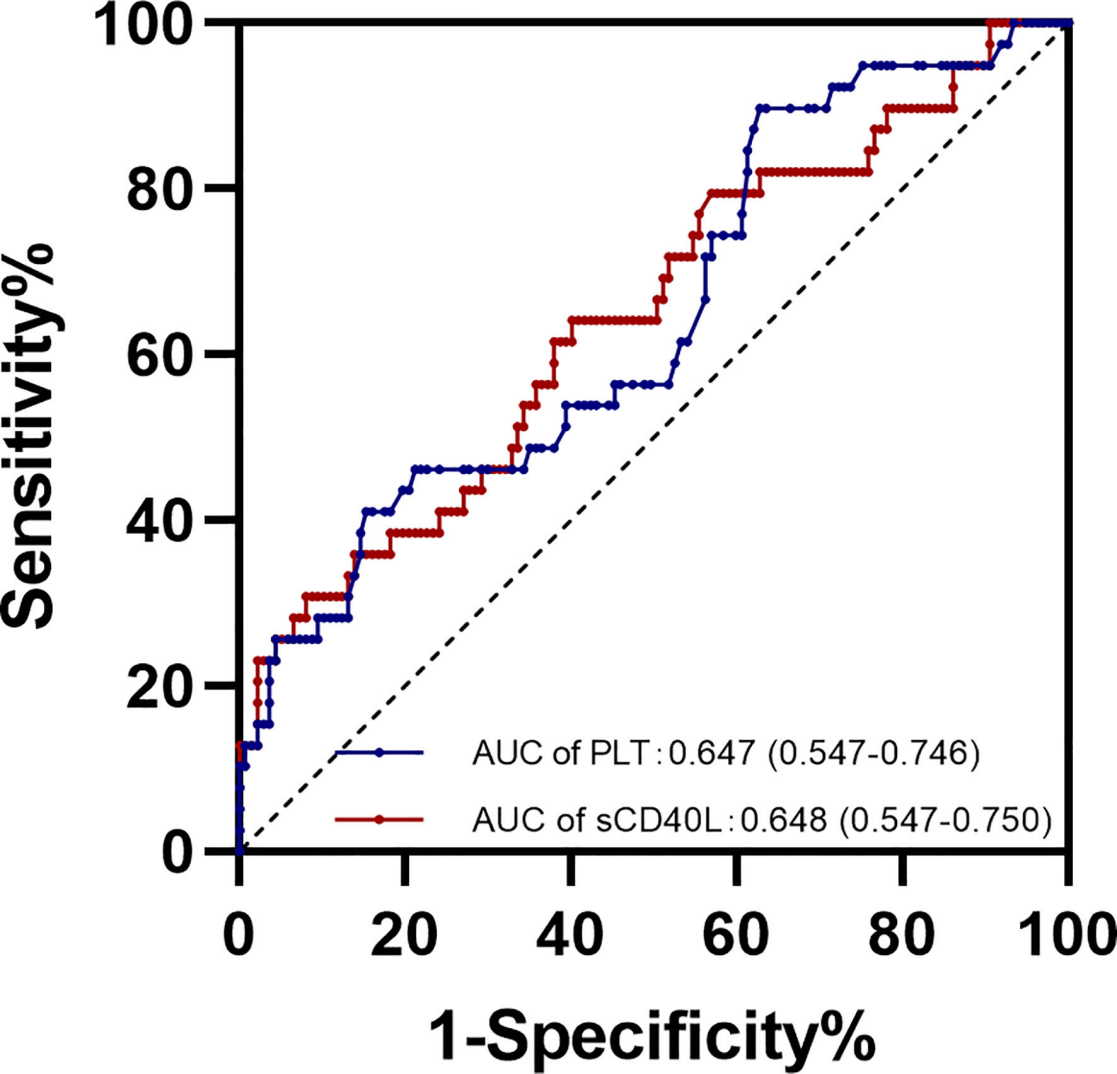
ROC curve for sCD40L and platelet levels in early pregnancy in predicting adverse neonatal outcomes.

Receiver operator characteristic (ROC) curve analysis was performed for the diagnostic performance of sCD40L and platelet levels for overall adverse neonatal outcomes (**Figure 3**). The best cutoff value for sCD40L and for platelet in predicting adverse neonatal outcomes was 5.625 ng/ml with an average area under curve (AUC) value of 0.648 (95% CI = 0.547 to 0.750) and 207 × 10^9^/L with an average AUC 0.647 (95% CI = 0.547 to 0.746), respectively.

### Multivariate logistic regression analysis of adverse neonatal outcomes in euthyroid pregnant women with positive TPOAb

**Figure 4:**
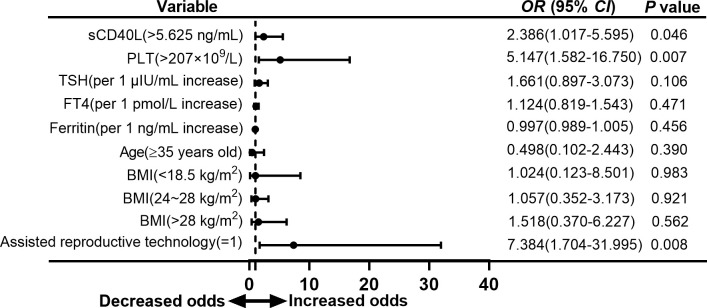
Forest plot of multivariate logistic regression analysis of risk factors for adverse neonatal outcomes in euthyroid pregnant women with positive TPOAb.

As we unexpectedly found that sCD40L, platelet counts, and the proportion of ART received were significantly higher in the group with adverse neonatal outcome, we attempted to evaluate the association of these risk factors with adverse neonatal outcome in euthyroid women with positive TPOAb. Our results revealed that serum sCD40L levels higher than 5.625 ng/ml and receiving ART were the independent risk factors for overall adverse outcome in euthyroid pregnant women with positive TPOAb in the first trimester (OR = 2.386, 95% CI = 1.017 to 5.595, *P* = 0.046; and OR = 7.384, 95% CI = 1.704 to 31.995, *P* = 0.008; **Figure 4**).

**Figure 5:**
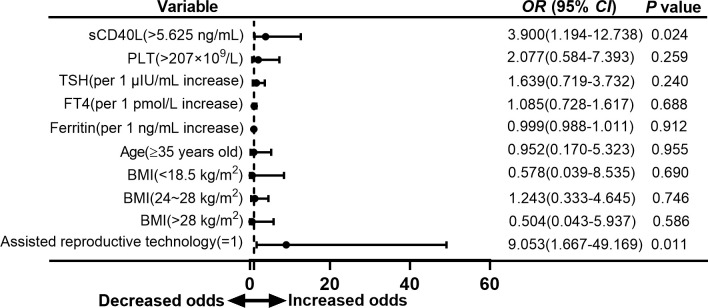
Forest plot of multivariate logistic regression analysis of risk factors for preterm birth in euthyroid pregnant women with positive TPOAb.

**Figure 6:**
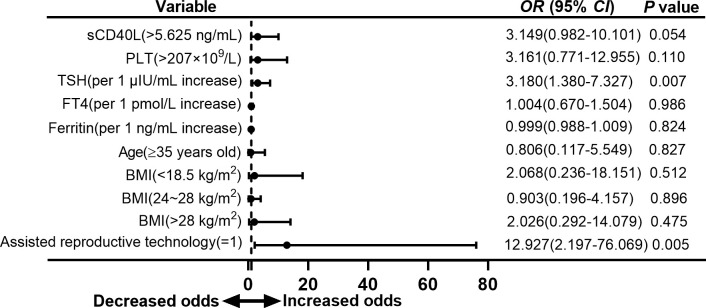
Forest plot of multivariate logistic regression analysis of risk factors for low birth weight in euthyroid pregnant women with positive TPOAb.

To analyze the association of risk factors and types of adverse neonatal outcome separately, we found that sCD40L level higher than 5.625 ng/ml was also a risk factor for preterm birth with an OR value of 3.900 (95% CI =1.194 to 12.738, *P* = 0.024; **Figure 5**). In the group with low birth weight, OR was 3.149 (95% CI = 0.982 to 10.101, *P* = 0.054; **Figure 6**). The OR values of receiving ART for preterm birth were 9.053 (95% CI = 1.667 to 49.169, *P* = 0.011; **Figure 5**) and 12.927 (95% CI = 2.197 to 76.069, *P* = 0.005; **Figure 6**) for low birth weight.

TSH was found to be risk factor for low birth weight (OR = 3.180, 95% CI = 1.380 to 7.327, *P* = 0.007; **Figure 6**) but showed no significant difference in overall adverse neonatal outcome and preterm birth.

Platelet counts higher than 207 × 10^9^/L were also a risk factor for overall adverse neonatal outcome in our study with an OR value of 5.147 (95% CI = 1.582 to 16.750, *P* = 0.007; **Figure 4**). However, multivariate logistic analysis did not show significant difference in group of preterm birth and low birth weight (**Figures 5**, **6**).

## Discussion

In the present study, a total of 39 (22.16%) euthyroid pregnant women with positive TPOAb had adverse neonatal outcomes during pregnancy. Preterm birth, low birth weight, and fetal macrosomia are the most common comorbidities. We found that serum sCD40L was significantly higher in the group with adverse neonatal outcome. Logistic regression analysis showed that sCD40L > 5.625 ng/ml in early pregnancy was independent risk factor for overall adverse neonatal outcome group and preterm birth. We proposed that the measurement of sCD40L in early pregnancy might have predictive value for adverse neonatal outcomes in euthyroid pregnant women with positive TPOAb.

Maternal thyroid hormones are dispensable for the development of the fetus, especially before the 20th gestational week, because the thyroid gland in the fetus is not well developed (1, 2). It is well documented that subclinical hypothyroidism is associated with adverse fetal outcomes (3, 4, 16–18). It remains unclear whether isolated TPOAb positivity is associated with adverse neonatal outcomes. The literature has yielded different results regarding the association of isolated TPOAb positivity and adverse neonatal outcomes. In a longitudinal study of 7,641 euthyroid pregnant women, TPOAb positivity was associated with a low birth weight (6). Yuan et al. reported that TPOAb positivity was not associated with poor pregnancy or fetal outcome in euthyroid women but with a higher risk of low birth weight in female fetuses (18). Meta-analysis showed that TPOAb positivity was associated with a higher risk of preterm birth (5, 7, 19). In our study, we found that about one of the four euthyroid pregnant women with positive TPOAb might have adverse outcome. Preterm birth, low birth weight, and fetal macrosomia were the most common comorbidities.

There are several possible explanations for the association of isolated TPOAb positivity with adverse pregnancy outcomes. Relative insufficiency of thyroid hormones due to positive TPOAb and the presence of thyroid antibodies and potential autoimmune disorders were possible underlying mechanisms of TAI-related adverse pregnancy outcomes (7). Women with TPOAb positivity had higher TSH and lower FT4 levels than women with TPOAb negativity (20, 21). Because of the high demand in early pregnancy, subtle thyroid dysfunction might exist because of the defect of capacity to compensate for high demand in early pregnancy. We observed a similar incidence of adverse neonatal outcomes in euthyroid women with TAI. There was no significant difference in TSH and FT4 between groups with adverse neonatal outcomes and without adverse neonatal outcomes. Thus, uncompensated maternal thyroid function during pregnancy is not the only explanation for the association between TAI and adverse neonatal outcomes. Then, it was reported that TPOAb could be transported through the placenta to cause abnormal fetal thyroid function and even abnormal growth (22). We did not find any differences in the titers of TPOAb. Therefore, our data did not support the mechanism of TPOAb itself and adverse neonatal outcomes. Maternal autoimmunity disorder was the other explanation (23). The activation of T cells and its related cytokines was reported to be participated in the immune disorder in patients with TAI (9, 10). Increased sCD40L can be detected and positively correlated with antibodies in many autoimmune diseases (24–26). sCD40 was reported to be positively correlated with TPOAb in patients with autoimmune thyroid disease (12, 13). Increased sCD40L was found to be higher in positive TPOAb women with adverse neonatal outcome in our study.

CD40-CD40L is a pair of membrane molecules. Both belong to the tumor necrosis factor receptor family and play an important role in the immune inflammatory response (12, 13). sCD40L is shed from activated T cells, platelets, and tissue cells and enters body fluids. Similar to its membrane molecules, it can combine with CD40 on immune cells to transmit information and is closely related to the pathogenesis of immune-related diseases. Activated platelets can release sCD40L and promote inflammation, activate the vascular endothelium, induce the expression of cyclooxygenase 2, and synthesize prostaglandins (11). Platelet counts were found to be higher in autoimmune thyroid diseases (27). It has been reported that the platelet activation of pregnant women with preeclampsia, fetal growth restriction, and preterm birth is higher than that of normal pregnant women and nonpregnant women (28–30). Increased platelet counts in the first trimester were suggested to be useful in predicting of early fetal demise (31). Our study also found that platelets were higher in pregnant women in the adverse neonatal outcome group. In addition, we found that there was a positive correlation between sCD40L and platelet count in pregnant women with TAI. CD40-CD40L might be a bridge mediating the immune response between platelets and lymphocytes in TAI (32).

A total of 13 (7.3%) participants received ART in our study. Receiving ART was closely linked to the adverse neonatal outcome such as preterm birth and low birth weight in our study, which was consistent with previous report (8, 19, 33). Previous cohort or clinical trials showed that levothyroxine (LT4) intervention for euthyroid women with positive TPOAb did not improve the pregnancy outcome (34, 35). The specific mechanism and causal relationship need to be further investigated.

Another interesting finding was that TSH was a risk factor for low birth weight in isolated positive TPOAb women in our study (as shown in **Figure 6**). As previously discussed, hypothyroidism and subclinical hypothyroidism were associated with low birth weight. It was also reported LT4 therapy for SCH was beneficial to decrease the risk of low birth weight in subclinical hypothyroidism (36). However, LT4 therapy may not mandatory for positive TPOAb women with TSH > 2.5 mIU/L and TSH < 4.0 mIU/L according to the latest 2017 ATA guideline (14). In addition, there were a few studies concentrated on low birth weight and isolated positive TPOAb in euthyroid women. Thus, it remains unclear what the best TSH range for positive TPOAb women is and whether LT4 therapy was beneficial for these women in early pregnancy.

The strengths of our study were as follows. First, all participants enrolled and analyzed in our study were euthyroid according to 2017 ATA guidelines. Thus, the possible effect of thyroid dysfunction was eliminated. Second, we concentrated on the adverse outcomes of euthyroid pregnant women positive for TPOAb and found, for the first time, that costimulatory molecules had a predictive role for adverse outcomes in early pregnancy.

This study also has some limitations. First, the sample size for this longitudinal observational study was small. Larger cohort studies are needed for further investigation, and sCD40L throughout pregnancy needs to be investigated. Second, we did not have longitudinal data on thyroid hormones before and after pregnancy. Furthermore, the predictive value of the marker should be further validated in clinic.

## Conclusions

In our study, we found that one of the four euthyroid women with TPOAb positivity might have adverse neonatal outcomes during pregnancy. These patients need to be carefully monitored during pregnancy. Increased sCD40L levels in the first trimester might be potential biomarker for adverse neonatal outcomes in pregnant women positive for TPOAb.

## Data availability statement

The clinical data of the population used to support the findings of this study are available from the corresponding author upon request.

## Ethics statement

This study was approved by the Institutional Review Board of Suzhou Municipal Hospital affiliated to Nanjing Medical University. The patients/participants provided their written informed consent to participate in this study.

## Author contributions

XXC and QW proposed and designed the study and performed the statistical analysis work. HZ, QS and XGC collected data and followed up the participants. XXC drafted the manuscript. LC reviewed and edited the manuscript and funding acquisition. All authors contributed to the article and approved the submitted version.
